# Cytoreductive surgery for giant locally advanced intra-abdominal tumors in Uganda

**DOI:** 10.1093/jscr/rjac178

**Published:** 2022-05-24

**Authors:** Michael Okello, Julius Nuwagaba, Henry Ddungu, Fred Machyo Okuku

**Affiliations:** Department of Anatomy, Makerere University College of Health Sciences, Kampala, Uganda; Department of Surgery, Lubaga Hospital, Kampala, Uganda; Department of Surgery, Lubaga Hospital, Kampala, Uganda; Department of Global Health Security, Makerere University Infectious Disease Institute, Kampala, Uganda; Uganda Cancer Institute, Kampala, Uganda; Uganda Cancer Institute, Kampala, Uganda

**Keywords:** cytoreductive surgery, debulking, giant, advanced, case series, Uganda

## Abstract

Cytoreductive surgery is removal of tumor as much as possible when complete resection is impossible because of advanced disease. It is a management option for giant intra-abdominal tumors with pressure symptoms. We present three patients who underwent cytoreductive surgery for giant intra-abdominal tumors between May 2019 and November 2021. Patient 1 had a gastrointestinal stromal tumor (GIST) involving stomach, spleen and transverse colon. En bloc resection of the GIST with the involved viscera was done. Patient 2 had a liposarcoma measuring 25.8 × 19.6 × 15.3 cm infiltrating the stomach, spleen and the left hemidiaphragm. Involved viscera and liposarcoma were resected en bloc. Patient 3 had a liposarcoma measuring 40 × 35 × 12 cm and encasing the left ureter. Mass was excised together with part of the left ureter and left ureter reconstructed. Giant intra-abdominal tumors are rare. Involvement of adjacent structures may necessitate multivisceral resections with or without organ reconstruction.

## INTRODUCTION

Cytoreductive or debulking surgery is removal of tumor as much as possible when complete resection is not possible because of the advanced nature of the disease [1, 2]. It provides a good treatment option for patients with pressure symptoms due to giant locally advanced intra-abdominal tumors that may or may not have metastasis. This is often achieved by peritonectomy, en bloc resection of the giant tumor and the involved adjacent viscera where necessary. In doing so, the aim is to remove all affected viscera while leaving the normal viscera *in situ* [3, 4]. Although complete tumor removal may be hard to achieve since it requires removal of adjacent organs and or even stripping of abdominal and pelvic walls [3]. Cytoreductive surgery is important for reducing the tumor bulk and hence improves the quality of life and prolongs the life of cancer patients [5]. We present three patients who underwent cytoreductive surgery for giant locally advanced intra-abdominal tumors at Lubaga Hospital between May 2019 and November 2021. Lubaga Hospital in Kampala is the second oldest hospital in Uganda founded in 1899 by the Catholic Church missionaries. The hospital currently has a bed capacity of 275.

## CASE SERIES

### Patient 1

**Figure 1:**
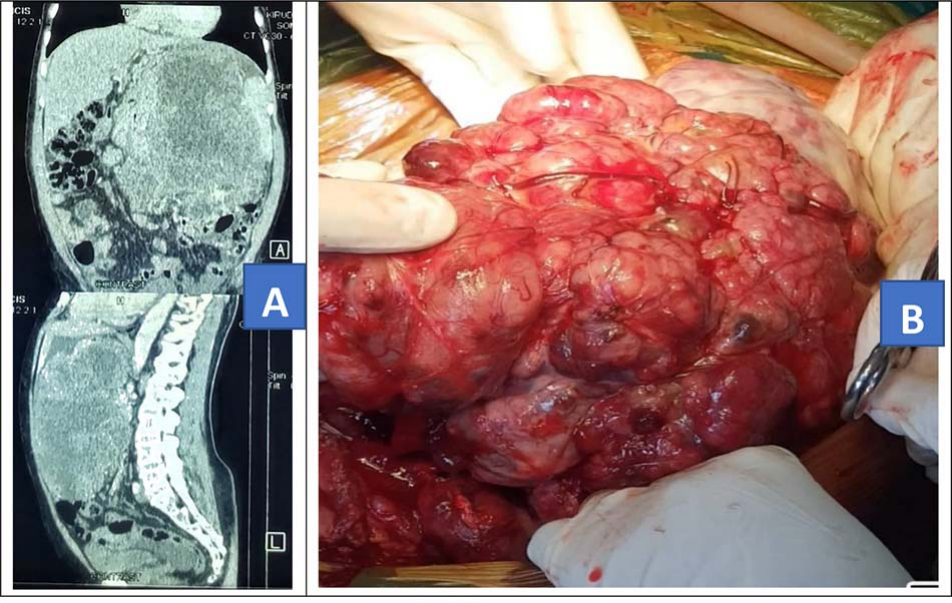
Contrasted abdominal CT scan (**A**) and intra-operative picture (**B**) for Patient 1.

A 72-year-old African male presented with 10-month history of abdominal mass associated with early satiety, epigastric discomfort and weight loss. He had no history of any chronic illness and had a vague memory of his father’s grandmother being diagnosed with possible nasopharyngeal carcinoma and his uncle had radiotherapy for unknown brain tumor. On examination, he had a grossly distended abdomen with a firm mass in the left upper quadrant which was mildly tender. Other examination findings were unremarkable. Abdominal computed tomography (CT) scan done prior to admission showed a giant solid retro gastric mass involving part of the greater curvature of the stomach, spleen and part of the distal one-third of the transverse colon (Fig. 1A). The patient was scheduled for open surgery.

Intra-operatively, a giant left upper quadrant tumor was found to be infiltrating and involving the spleen, greater curvature of the stomach and transverse colon (Fig. 1B). The tumor was mobilized from its retroperitoneal attachment, the splenic vessels were ligated and the entire tumor removed. Partial gastrectomy was done for the involved part and a 20-cm long distal transverse colectomy was done and a double barrel colostomy fashioned. The patient lost 1500 ml of blood and was transfused with two units of whole blood intra-operatively. The tumor was taken for histology and the results showed atypical pleomorphic spindle-shaped cells with abnormal mitosis. Immunohistochemistry: cells were positive for CD34 and CKIT which is in keeping with gastrointestinal stromal tumor.

Post-operatively, the patient was transfused with two more units of blood and was given antibiotics, epinephrine and analgesics. He also received subcutaneous enoxaparin 60 mg once a day and compression stockings as part venous thromboprophylaxis. The patient started oral sips on the fourth post-operative day, and the abdominal drain was removed on the fifth post-operative day. The patient was discharged on the 10th post-operative day and was referred to the Uganda cancer institute for further management; the oncology team started him on tabs imatinib mesylate 400 mg once a day. The patient improved and was readmitted 6 weeks later for colostomy reversal that was done successfully and the patient discharged after 8 days with no complications. The patient’s follow-up was unremarkable at 3 months. Repeat abdominal CT scan at 6 months, 1 year and 2 years showed suspected non increasing tumor recurrence along the lesser curvature of the stomach, and repeat upper gastrointestinal endoscopy at 2.5 years’ post-surgery was unremarkable. Patient was referred back to the oncology team who continued the patient on imatinib mesylate 400 mg once a day for another 6 months in order to complete 3 years of treatment**.**

### Patient 2

A 60-year-old African male was admitted with a 1-month history of gradual on set of abdominal pain and constipation. This was associated with early satiety, but there was no vomiting and no nausea. He had no history of weight loss. A week prior to presentation, the symptoms had intensified and was associated with abdominal distension. He had not passed stool in that week although was able to pass flatus. His systemic review was unremarkable. He was a known patient of hypertension on telmisartan/hydrochlorothiazide, and there was no other chronic illness and no past history of surgery. His mother reportedly underwent an amputation for possible osteosarcoma when he was still a child.

**Figure 2:**
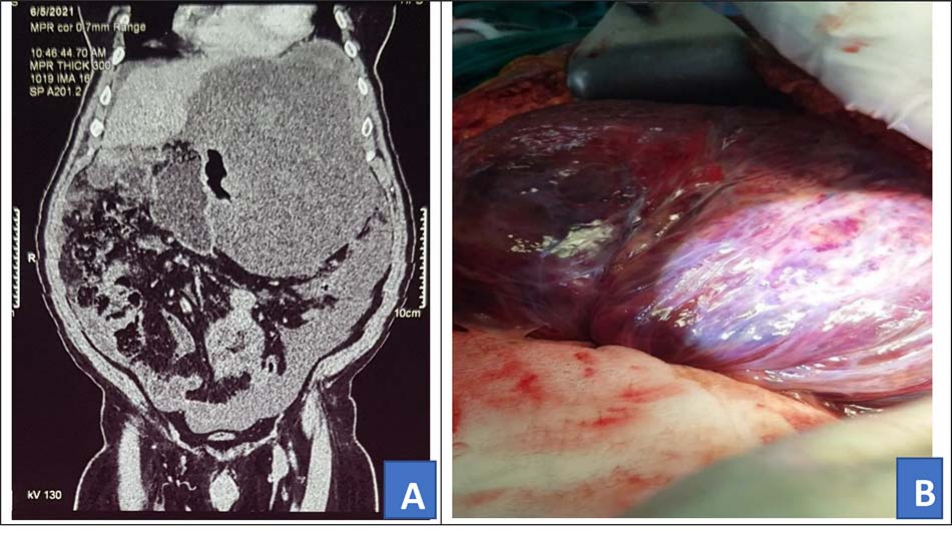
Preoperative abdominal CT scan (**A**) and intra-operative picture (**B**) for Patient 2.

On examination, he was fair, with a temperature of 36.4°C, a pulse rate of 72 beats per minute and had a blood pressure of 128/85 mmHg. The abdomen was grossly distended with a palpable mass in the left upper quadrant. He had no hepatomegaly, He had normal bowel sounds. Other systems’ exam was grossly normal. An abdominal CT was done and it showed a 25.8 × 19.6 × 15.3 cm mass in the left upper abdominal quadrant (Fig. 2A). The patient was scheduled for an exploratory laparotomy.

**Figure 3:**
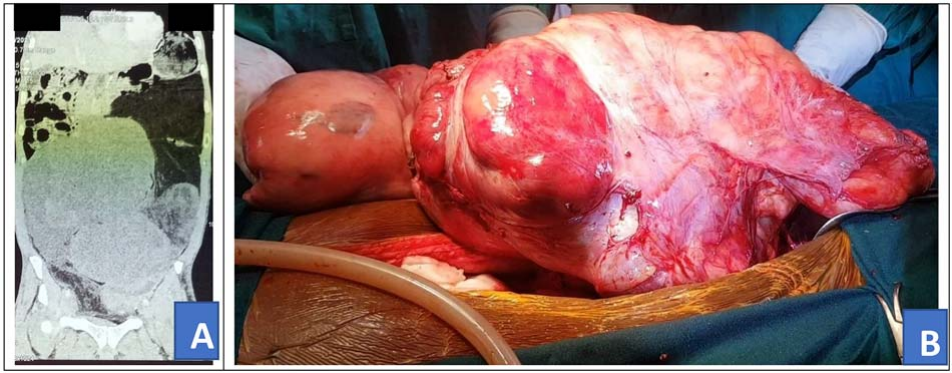
Preoperative abdominal CT scan (**A**) and intra-operative picture (**B**) for Patient 3.

Intra-operatively, a giant 28 × 30 cm retro gastric mucinous tumor was found involving the greater curvature of the stomach, the spleen and part of the left hemi diaphragm (Fig. 2B). The patient was also found to have moderate ascites of 500 ml. The tumor was mobilized and resected. In addition, the greater curvature of the stomach, part of the left hemi diaphragm and spleen were resected. Diaphragm was repaired with ethylon 1–0.

Post-operatively, the patient was managed with epinephrine, metronidazole, morphine, a fentanyl patch and IV normal saline. He was also given enoxaparin for thromboprophylaxis. On the second post-operative day, the patient developed a pleural effusion and was put on chest physiotherapy with an incentive spirometer and a chest tube inserted. The patient started ambulating and soft diet on the fourth post-operative day. The patient however developed wound dehiscence on Day 7 after operation and this was managed with daily dressing and nadifloxacin cream alongside other medication. The chest tube was removed on the 12th post-operative day. The patient was discharged on the 15th post-operative day without any further complications. Histology noted 7-kg mass with atypical spindle cells, multivacuolated lipoblasts. Immunohistochemistry: cells were focally positive for S 100, which was suggestive of liposarcoma. Follow-up review at 2 weeks and 1 month were unremarkable. Patient was referred to Uganda cancer institute for further management but became lost to follow-up and later returned after 6 months and reported to have been on herbal medication during this period. Clinical evaluation and repeat abdominal ultrasound (US) scan at 6 months were unremarkable. He was then referred to Uganda Cancer institute for further management.

### Patient 3

A 46-year-old African male was admitted with history of progressive weight loss and abdominal distension for 7 months. He had no history of abdominal pain, diarrhea of constipation. He, however, reported a history of on-and-off evening fevers. In review of systems, he had a history of swelling of left lower limb and had been on tabs rivaroxaban 20 mg once daily as treatment for deep venous thrombosis diagnosed 5 months prior. Rivaroxaban was stopped 24 hours prior to surgery and then continued post-operatively to complete 6 months of treatment. On examination, he had gross abdominal distension with a firm mass arising from the left lower quadrant. He also had swelling of the left lower limb with darkening of the overlying skin. Other systemic examinations we unremarkable. Prior to admission, he had done an abdominal CT scan that showed a huge mixed density soft tissue intra-abdominal mass exerting pressure on surrounding viscera (Fig. 3A). Fibrosarcoma diagnosis had been confirmed by US-guided biopsy and histology. The patient received neo-adjuvant chemotherapy, intravenous doxorubicin 100 mg with intravenous mesna 1000 mg in 100 ml of normal saline and intravenous ifosfamide 9500 mg in 3 l of normal saline with intravenous mesna 2375 mg in 1 l of normal saline every 21 days, four cycles of chemotherapy prior to referral for surgery. Investigations, including complete blood count, renal function tests, liver function tests and prothrombin time, were all grossly normal and the patient was scheduled for cytoreductive tumor resection.

**Figure 4:**
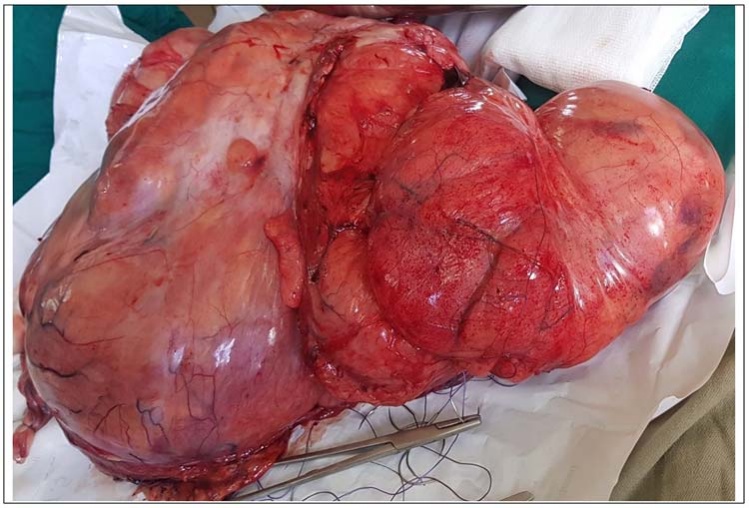
Tumor removed from Patient 3.

Intra-operatively, a giant 40 × 35 × 12 cm liposarcoma measuring 10.1 kg was found involving the left ureter (Fig. 3B). The transverse colon, descending colon and sigmoid colon were attached to the anterior part of the mass and the rectum was displaced to the right. Other abdominal viscera were normal. The mass was mobilized from the colon. The entire mass en bloc, including the left ureter, were excised (Fig. 4). Then, the left to right ureteroureterostomy with a Double-J stent insertion done.

Post-operatively, the patient was managed with antibiotic, epinephrine, metronidazole and analgesics, morphine and a fentanyl patch. The patient started oral sips on the first post-operative day, soft diet on the second post-operative day. On the fifth post-operative day, the patient underwent a re-laparotomy due to leakage of the left to right ureteroureteric anastomosis. Leaked anastomosis was dismantled, previous anastomotic site was resected and right end-to-end spatulated uretero-ureteric anastomosis was done with double J stent *in situ*. A 15-cm terminal ileal conduit was isolated while maintaining its vascular connection on from the mesentery proper. Distal ileum stump was closed off, end to side ileo-ascending anastomosis was created to maintain intestinal continuity, proximal end of the isolated ileal conduit was closed off, end to side left uretero-ileostomy was created and distal end of the isolated ileal conduit was anastomosed to the dome of the urinary bladder. Hemostasis was achieved, mops and instrument count were done and abdomen was closed in layers with a pelvic drain *in situ*. Histology showed a dedifferentiated liposarcoma. The patient was discharged on the 12th day after the first surgery with abdominal drain *in situ* without further complications. Review after 1 week, drain was removed. Double J stent was removed after 8 weeks and patient referred to Uganda cancer institute for further management.

## DISCUSSION

Giant intra-abdominal tumors exert a lot of pressure symptoms and this can be uncomfortable just like in our patient’s case [6]. These tumors present with complications, such as in Patient 3, who had lower limb swelling with deep venous thrombosis from the tumor’s pressure on the left common iliac vein. Given the symptomatic nature of these giant tumors and a high risk of metastasis, these tumors have a high morbidity and mortality rate with or without surgery. Successful treatment of these malignancies requires a multidisciplinary approach as seen in the management of these three patients. The chemotherapy may be neoadjuvant, intra-operative as done during hyperthermic intraperitoneal chemotherapy (HIPEC) or adjuvant for better tumor eradication [3, 7]. Cytoreductive surgery involves complete mobilization of the tumor and resection of involved adjacent organs with the ultimate aim of ensuring complete macroscopic resection of disease [8, 9]. It is important to screen patients who are fit for surgery to improve outcomes [3, 10]. Extra-abdominal metastasis is a relative contraindication for cytoreductive surgery [11]. An overall performance status of the patient as well as optimal control of all systemic illness is also necessary before carrying out such major operation and all the three patients in discussion had been fully screened for this [12].

Complete macroscopic tumor removal requires removing adjacent viscera with evidence of disease, such as partial gastrectomy in Patient 1; resection of greater curvature of the stomach, the spleen and part of the left hemi diaphragm in Patient 2 and left ureter in Patient 3 [7, 8].

Cytoreductive surgery associated with multivisceral resection of involved adjacent organs or structures with or without reconstruction remains a complex procedure, which is safe in expert hands as seen in the cases of these three patients. Complications like anastomotic leakage and fistula may occur and these present high morbidity and mortality [13], but early detection and intervention is key in achieving good outcomes as seen in Patient 3 who got an anastomotic leakage. Other complications include long operative time and blood loss [9]. HIPEC, peritonectomy and cytoreductive surgery combination is a currently recommended treatment option for management of giant abdominal tumors and peritoneal carcinomatosis and should be encouraged as it improves the survival time of cancer patients [14, 15]. However, all the three patients discussed in this case series did not have peritoneal carcinomatosis, so none of them underwent HIPEC or peritonectomy. We were able to do peritonectomy, but HIPEC is currently not available in Uganda.

## CONCLUSION

Giant intra-abdominal tumors are rare and when they occur, they may involve adjacent structures, thereby necessitating multivisceral resections in order to ensure complete macroscopic resection with or without organ reconstruction.

## CONFLICT OF INTEREST STATEMENT

None declared.

## STATEMENT OF ETHICS

Written informed consent was obtained from all the three patients for publication of this case series and accompanying images. A copy of the written consent is available for review by the editor-in-chief of this journal on request.
